# 2439. Excess Length of Stay and Mortality among the Critically Ill Adult Patients with Catheter-Associated Urinary Tract Infections in a Tertiary Care Teaching Hospital in Nepal

**DOI:** 10.1093/ofid/ofad500.2058

**Published:** 2023-11-27

**Authors:** Sailesh K Shrestha

**Affiliations:** National Academy of Medical Sciences, Bir Hospital, Lalitpur, Bagmati, Nepal

## Abstract

**Background:**

Patients in the Intensive Care Units (ICU) with Catheter-associated Urinary tract infections (CAUTI) stay longer in the hospital and have excess mortality. Conventional techniques like matching designs or regression analyses to obtain these estimates are prone to time-dependent bias and bias due to competing events. Instead, a survival analysis technique called multistate modeling that describes the occurrence of events as transitions between multiple states over time, has been recommended. Reliable estimates of excess length of stay and mortality among patients with CAUTIs, using appropriate methodologies, have not been reported from Nepal. In this study, we examined the mortality and length of stay that could be attributed to CAUTIs in the ICU of our center.

**Methods:**

We conducted this prospective cohort study in the 8-bedded medical ICU of a 458 bedded tertiary care teaching hospital in Kathmandu, Nepal. We included all the patients aged ≥ 16 years admitted to the medical ICU and with an indwelling urinary catheter for more than two calendar days during the one-year study period from June 2021 to July 2022. We diagnosed CAUTI using US CDC definitions and determined the antibiotic susceptibility of the bacteria when isolated. We summarized the categorical variables with frequency and numerical variables with median (interquartile range [IQR]). With the assumption of a constant hazard model, we used multistate models to calculate the transitional probabilities and estimate the excess length of stay and mortality.

**Results:**

Of the 209 patients receiving urinary catheterization, more than half of them (120, 57.42%) were males and the median age was 50 (IQR 38 – 67.25) years. There were 32 (15.3%) CAUTI events identified. The urine samples of 25 patients yielded 27 microorganisms; 20 isolates among them were multi-drug resistant isolates. *Escherichia coli* was the most common microorganism. Overall, the patients with CAUTI stayed 2.5 days longer in the ICU and had 13% higher mortality than those without CAUTI.

**Conclusion:**

The high burden of CAUTIs and the associated excess length of stay and mortality provide an approximation of the number of lives that could be saved and the number of bed days that could be gained by the appropriate CAUTI preventive and management strategies and should be reinforced.

**Disclosures:**

**All Authors**: No reported disclosures

